# Global Dosage Compensation Is Ubiquitous in Lepidoptera, but Counteracted by the Masculinization of the Z Chromosome

**DOI:** 10.1093/molbev/msx190

**Published:** 2017-07-06

**Authors:** Ann Kathrin Huylmans, Ariana Macon, Beatriz Vicoso

**Affiliations:** 1 Institute of Science and Technology Austria, Klosterneuburg, Austria

**Keywords:** sex chromosome evolution, sex-biased gene expression, faster-Z, tissue-specific expression, gonads, female-heterogamety

## Abstract

While chromosome-wide dosage compensation of the X chromosome has been found in many species, studies in ZW clades have indicated that compensation of the Z is more localized and/or incomplete. In the ZW Lepidoptera, some species show complete compensation of the Z chromosome, while others lack full equalization, but what drives these inconsistencies is unclear. Here, we compare patterns of male and female gene expression on the Z chromosome of two closely related butterfly species, *Papilio xuthus* and *Papilio machaon*, and in multiple tissues of two moths species, *Plodia interpunctella* and *Bombyx mori*, which were previously found to differ in the extent to which they equalize Z-linked gene expression between the sexes. We find that, while some species and tissues seem to have incomplete dosage compensation, this is in fact due to the accumulation of male-biased genes and the depletion of female-biased genes on the Z chromosome. Once this is accounted for, the Z chromosome is fully compensated in all four species, through the up-regulation of Z expression in females and in some cases additional down-regulation in males. We further find that both sex-biased genes and Z-linked genes have increased rates of expression divergence in this clade, and that this can lead to fast shifts in patterns of gene expression even between closely related species. Taken together, these results show that the uneven distribution of sex-biased genes on sex chromosomes can confound conclusions about dosage compensation and that Z chromosome-wide dosage compensation is not only possible but ubiquitous among Lepidoptera.

## Introduction

In many species, sex is genetically determined via sex chromosomes that have evolved from a pair of autosomes (Rice 1984). After the initial acquisition of a sex-determining gene, recombination between the sex chromosomes is often suppressed to preserve linkage of alleles with sex-specific benefits and the sex-determining region (reviewed in Bergero and Charlesworth 2009). In the absence of recombination, purifying selection on the sex-specific chromosomes becomes inefficient, and Y or W chromosomes accumulate deleterious mutations and eventually degenerate via pseudogenization (reviewed in Charlesworth etal. 2005; Bachtrog 2006). The loss of Y/W genes poses a problem for the heterogametic sex, as gene dosage is correlated with gene copy number, and haploidy of the X/Z can cause imbalances in gene networks that involve both sex-linked and autosomal genes (Jeong etal. 2001; Mank 2009). Dosage compensation mechanisms, that is, mechanisms that target the X/Z chromosome and regulate its expression, often evolve to overcome this imbalance (Charlesworth 1978) and have been studied extensively in species with XY sex determination, such as mammals, *Drosophila*, and *Caenorhabditis elegans*. While dosage compensation has evolved independently in these clades, and differs in its molecular mechanism, the result is the same: full equalization of X-linked gene expression between the sexes (reviewed in Mank 2013).

Species with ZW sex determination, where females are the heterogametic sex, show a different pattern: in birds (Ellegren etal. 2007; Itoh etal. 2007; Mank and Ellegren 2009), *Schistosoma* (Vicoso and Bachtrog 2011), fish (Chen S etal. 2014), and snakes (Vicoso, Emerson, etal. 2013), dosage compensation does not affect the whole chromosome and instead only a few dosage-sensitive genes seem to be compensated via individual regulation (Naurin etal. 2010; Bachtrog etal. 2011; Mank 2013). Why there should be such a difference between XY and ZW systems is unclear, although several hypotheses have been put forward:

1. Differences in dosage sensitivity between the sexes (if females are less sensitive then chromosome-wide Z compensation may be unnecessary; Mank 2009) or between clades (in this case, the apparent XY-dosage compensation association could disappear with increasing sample size; Graves and Disteche 2007).

2. Sexual antagonism: selection is predicted to favor the accumulation of mutations with male-specific benefits on Z chromosomes, potentially leading many Z-linked genes to acquire male-specific functions (Albert and Otto 2005).

3. Stronger selection in males: the initial acquisition of dosage compensation likely involves mutations that up-regulate Z-linked expression in both sexes, and which are favored in the heterogametic sex but disfavored in the homogametic sex (Mullon etal. 2015). Since males are often under stronger selective pressures due to sexual selection, such mutations are more likely to be fixed in male-heterogametic systems (Mullon etal. 2015).

4. Reduced efficacy of selection on the Z chromosome: Males typically have larger variance in reproductive success than females, which strongly reduces the effective population size of the Z and reduces the probability that beneficial compensatory mutations will be fixed there (Mullon etal. 2015).

These hypotheses are not exclusive, and understanding the extent to which they contribute to the differences between female- and male-heterogametic species is an ongoing challenge.

The last ZW clade that has been studied in detail is the insect order Lepidoptera (moths and butterflies). In early expression studies, Z-linked genes were individually found to be uncompensated (Goldschmidt 1921; Stehr 1958; Johnson and Turner 1979). Lack of dosage compensation was initially supported by a large-scale expression study in the silkworm *Bombyx mori* (Zha etal. 2009); however, a reanalysis of that and additional data showed that this was due to normalization issues, and that the Z was in fact globally equalized between the sexes (Walters and Hardcastle 2011). It is now known that a mechanism of chromosome-wide compensation is indeed present in the silkworm, and is dependent on the sex-determination pathway (Kiuchi etal. 2014), similar to XY species. Chromosome-wide compensation has similarly been detected in the moths *Manduca sexta* (Smith etal. 2014) and *Cydia pomonella* (Gu etal. 2017) and in two *Heliconius* butterfly species (Walters etal. 2015). On the other hand, the Indian mealmoth *Plodia interpunctella*, shows no evidence of chromosome-wide equalization of expression (Harrison etal. 2012). Hence, while it is clear that Lepidoptera differ from other ZW clades in that they can have fully compensated Z chromosomes, the extent to which this happens appears to vary, making them an interesting clade in which to study the acquisition of global versus local compensation. Full dosage compensation is defined here as equal levels of gene expression between the sexes throughout the Z chromosome (global) rather than individual regulation at the level of each gene (local). An added complication is that studies in different Lepidoptera species have used a variety of tissues, composite structures, and developmental stages, as well as different methods to assess gene expression (microarrays, RNA-sequencing) often making direct comparisons difficult (Gu etal. 2017).

In this paper, we compare dosage compensation patterns between Lepidoptera species at two different scales. First, we focus on two closely related butterfly species, *Papilio xuthus* and *Papilio machaon*, which diverged <35 Ma (Zakharov etal. 2004). We find that even within a genus, patterns of Z-linked expression can vary, but that this is due to shifts in the distribution of genes with strongly sex-biased expression rather than to differences in equalization between the sexes. We also obtained and analyzed RNA-seq data from multiple tissues of two distantly related moth species in which dosage compensation had been studied before and found to differ, *P. interpunctella* and *B. mori*. We show that the difference between the two species is caused by strong male-biased expression of the Z chromosome in the gonads, as previously suggested (Arunkumar etal. 2009; Gu etal. 2017), and that full dosage compensation is observed once this has been accounted for. Z chromosome-wide dosage compensation is therefore more widespread in Lepidoptera than previously thought, and the male-biased expression that is observed in some species appears to be instead a consequence of the functional masculinization of the Z chromosome.

## Results

### Variable Patterns of Z-Linked Expression in Closely Related *Papilio* Species

**Fig. 1.**
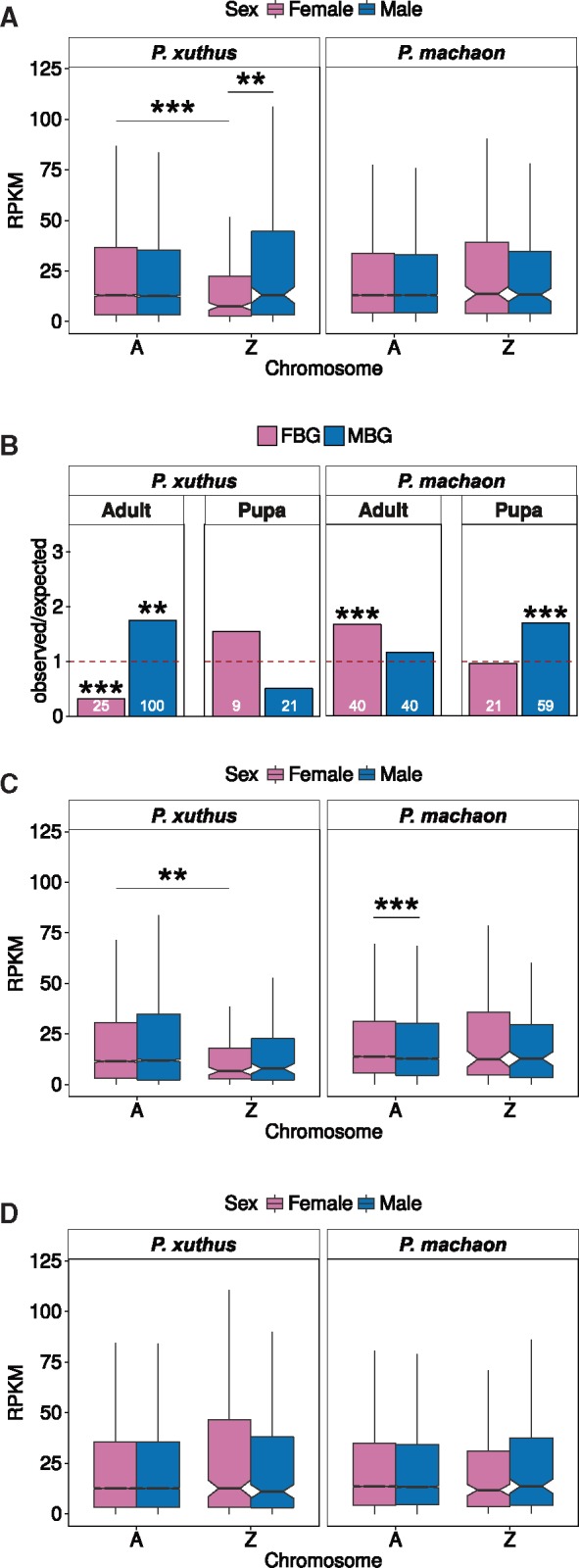
Dosage compensation in *Papilio* butterfly species for expressed genes (RPKM > 0 in both sexes). (*A*) Adult expression on the Z chromosome and the autosomes compared in males and females. (*B*) Distribution of sex-biased genes on the Z chromosome calculated as the number of observed genes over the number of expected ones. Absolute gene numbers are stated in bars. (*C*) Adult expression on the Z chromosome and the autosomes compared in males and females excluding sex-biased genes. (*D*) Pupa expression on the Z chromosome and the autosomes compared in males and females. ***P <* *0.001, **P <* *0.01, *P <* *0.05, comparisons without significance stars are nonsignificant (*P *>* *0.05), Wilcoxon rank test for RPKM values, FET for over-/underrepresentation of sex-biased genes.

We used a recently published RNA-sequencing (RNA-seq) data set for male and female adults and pupae to compare patterns of Z-linked gene expression between two closely related butterfly species, *P. machaon* and *P. xuthus* (Li etal. 2015). Genes were classified as Z-linked or autosomal according to the *P. xuthus* linkage map (Li etal. 2015) combined with a reciprocal best hit mapping approach to detect 1:1 orthologs between the two species. This resulted in 12,726 autosomal and 290 Z-linked genes in *P. xuthus* and 10,580 autosomal and 235 Z-linked ones in *P. machaon*. All main figures show expression patterns in males and females using only genes that were expressed in both sexes (RPKM > 0), but results are robust to different RPKM cut-offs (supplementary figs. S1–2, S5–6, S8–9, Supplementary Material online). In *P. xuthus* adults, gene expression patterns are consistent with absence of global dosage compensation: there is a significant reduction in Z chromosome expression in females relative to males (*P* value = 0.0021, M:F = 1.748) and the expression of the Z chromosome is significantly lower than that of the autosomes in females (*P* value = 0.0002, fig. 1*A*, Z:A = 0.581, table 1). In *P. machaon* on the other hand, expression patterns are consistent with complete Z compensation with no differences between the sexes for Z chromosome expression and similar expression of Z-linked and autosomal genes in females (*P* values > 0.5, fig. 1*A*, M:F = 1 and Z:A = 1, table 1). These results are consistent across a range of different RPKM cut-offs (supplementary figs. S1 and S2, Supplementary Material online). In pupae, full Z chromosome compensation is present of both species (*P* values > 0.29; fig. 1*D* and table 1) for all RPKM cut-offs.

**Table 1.** Average Expression Ratios for Males and Females on the Z Chromosome and the Autosomes Across Multiple Lepidoptera Species and Tissues and Stages and the Effect of Sex-Biased Genes (SBG).

Species	Stage	Tissue	SBG Included	Z:A Males	Z:A Females	M:F on A	M:F on Z
*Papilio xuthus*	Adult	Whole body	Yes	1.043	0.581	0.973	1.748
*P. xuthus*	Adult	Whole body	No	0.657	0.578	1.045	1.187
*P. xuthus*	Pupa	Whole body	Yes	0.880	0.999	1.000	0.881
*P. machaon*	Adult	Whole body	Yes	1.015	1.044	1.000	0.972
*P. machaon*	Adult	Whole body	No	0.996	0.909	0.925	1.014
*P. machaon*	Pupa	Whole body	Yes	1.022	0.869	0.984	1.158
*Bombyx mori*	Adult	Gonads	Yes	0.930	0.641	0.994	1.441
*B. mori*	Adult	Gonads	No	0.845	0.858	0.992	0.977
*B. mori*	Adult	Thorax	Yes	0.858	0.789	1.008	1.096
*B. mori*	Adult	Heads	Yes	0.833	0.810	0.985	1.013
*Plodia interpunctella*	Adult	Gonads	Yes	0.882	0.499	0.967	1.709
*P. interpunctella*	Adult	Gonads	No	0.664	0.560	0.972	1.151
*P. interpunctella*	Adult	Thorax	Yes	0.895	0.910	0.992	0.976
*P. interpunctella*	Adult	Heads	Yes	0.772	0.836	0.999	0.922
*P. interpunctella*	Larva	Heads	Yes	0.757	0.746	1.003	1.017

### Sex-Biased Genes on Sex Chromosomes Can Confound Patterns of Dosage Compensation

The distribution of genes with sex-biased or sex-specific expression often varies between autosomes and sex chromosomes (Ellegren and Parsch 2007, and see “Discussion” section), and can lead to overall sex-biased expression of the X/Z chromosomes even in the presence of dosage compensation. We tested whether the chromosomal distribution of strongly sex-biased genes (>2-fold difference between males and females, a difference unlikely to be caused by haploidy of the Z) was different between the two *Papilio* species, and whether this accounted for the apparent difference in expression equalization between the sexes. We found that, in *P. xuthus* adults, the Z chromosome was enriched for male-biased genes (MBG) and depleted for female-biased genes (FBG), while in *P. machaon* adults, FBG were overrepresented on the Z chromosome and MBG were distributed as expected by chance (fig. 1*B*). The number of sex-biased genes on the Z chromosome in *P. xuthus* pupae was low (21 MBG and 9 FBG) and no enrichment or depletion on the Z chromosome could be observed. *Papilio machaon* pupae showed an enrichment of MBG on the Z chromosome (fig. 1*B*). These patterns also held when the cut-off used to define sex-biased genes was increased to a fold-change > 4 (supplementary fig. S3, Supplementary Material online).

Excluding genes with strongly sex-biased expression from our dosage compensation analysis (both on the Z chromosome and the autosomes) removed most differences in Z-linked gene expression between males and females (fig. 1), suggesting that these strongly biased genes were responsible for the disparity between the species. While the *P. xuthus* Z chromosome remained expressed at lower levels in females than the autosomes after excluding sex-biased genes (*P* value < 0.01, fig. 1*C*, Z:A = 0.578, table 1), the difference between males and females could no longer be detected (*P* value > 0.7, Z:A = 0.657 in males, table 1). This result held independent of the chosen RPKM cut-off (supplementary fig. S1, Supplementary Material online).

In *P. machaon* adults, the exclusion of sex-biased genes did not affect the results for the Z, but led to higher expression of autosomal genes in females than in males (*P* value = 5.0e-7, fig. 1*C*). The removal of sex-biased genes did not affect the patterns for pupae of either species (supplementary figs. S1 and S2, Supplementary Material online). Furthermore, the Z chromosome in *P. xuthus* is not entirely conserved as parts of it are homologous to *B. mori* chromosome 17 and chromosome 5 (Li etal. 2015). In order to exclude the possibility that the dosage compensation patterns in *Papilio* are driven by these evolutionary younger parts that may behave more like autosomes, we reanalyzed our data using only Z-linked genes that have 1-to-1 orthologs on the *B. mori* Z chromosome. Our results concerning dosage compensation with and without sex-biased genes and the enrichment of sex-biased genes on the Z chromosome are exactly the same as when we use all Z-linked genes (supplementary fig. S4, Supplementary Material online). Hence, the inclusion of newer parts of the Z chromosome does not affect our inference about dosage compensation in *Papilio*. These results show that the enrichment of MBG and depletion of FBG on the *P. xuthus* Z chromosome biases the inferences about dosage compensation, and that Z-linked genes are likely compensated once strong sex-biased expression has been accounted for.

### Fast Evolution of Expression of Z-Linked and Sex-Biased Genes

We investigated whether an increased rate of evolution of Z-linked and/or sex-biased genes (reviewed in Ellegren and Parsch 2007; Parsch and Ellegren 2013) might have contributed to the striking difference in the sex-biased gene content of the Z chromosome of the sister species *P. xuthus* and *P. machaon*. Genes are expected to evolve faster on the Z chromosome than the autosomes because: 1) The low effective population size of the Z chromosome allows for the fixation of many slightly deleterious mutations (Mank, Nam, etal. 2010; Mank, Vicoso, etal. 2010); 2) Selection in hemizygous females favors the fixation of new beneficial recessive mutations on the Z (Charlesworth etal. 1987; Meisel and Connallon 2013). “Faster-Z” evolution of coding sequence and gene expression has been detected in birds (Mank etal. 2007; Mank, Nam, etal. 2010; Corl and Ellegren 2012; Dean etal. 2015; Wright etal. 2015), but results have been inconsistent in Lepidoptera (Sackton etal. 2014; Rousselle etal. 2016); both compared only sequence evolution.

Sex-biased genes also show high rates of turnover and sequence divergence in various clades (Meiklejohn etal. 2003; Zhang etal. 2007; Assis etal. 2012; Harrison etal. 2015; Grath and Parsch 2016; Papa etal. 2016), likely due to the action of sexual selection on mating traits (Harrison etal. 2015; Grath and Parsch 2016), but whether this pattern holds in Lepidoptera is unknown.

**Fig. 2.**
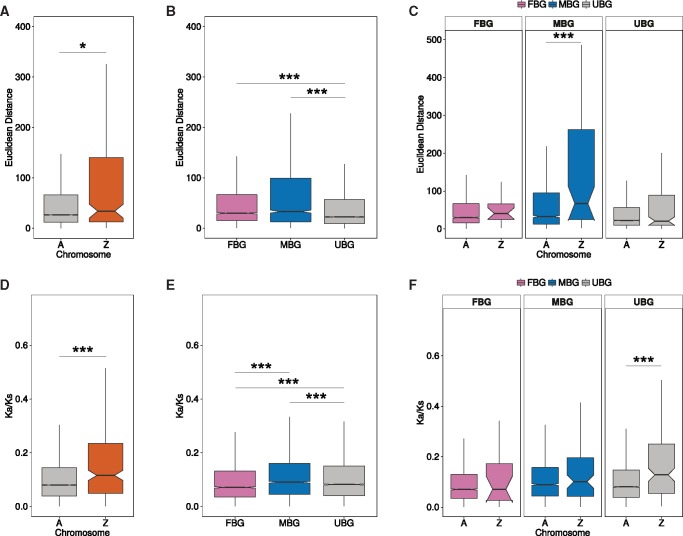
Evolutionary rates of sex-biased genes and Z-linked genes of *Papilio* butterflies. Expression divergence: (*A*) Euclidean distance in expression for autosomal and Z-linked genes. (*B*) Euclidean distance in expression for male-biased, female-biased, and unbiased genes. (*C*) Euclidean distance in expression for sex-biased genes and chromosomal location (Z chromosome vs autosomes). Sequence divergence: (*D*) Ka/Ks ratios for autosomal and Z-linked genes. (*E*) Ka/Ks ratios for sex-biased genes. (*F*) Ka/Ks ratios for for sex-biased genes and chromosomal location. Classification of sex-biased genes is based on *P. xuthus* adults. ***P <* *0.001, **P <* *0.01, *P <* *0.05, comparisons without significance stars are nonsignificant (*P *>* *0.05), Wilcoxon rank test.

We estimated expression divergence (as Euclidean distances), as well as sequence divergence (as Ka/Ks values), for all 1-to-1 *P. xuthus*–*P. machaon* orthologs with a known chromosomal location (10,803 genes). When expression levels were considered, Z-linked genes showed significantly larger divergence than autosomal ones (*P* value = 0.01, fig. 2*A*). Furthermore, genes with male-biased (MBG) and female-biased (FBG) expression in *P. xuthus* adults showed stronger expression divergence than unbiased genes (UBG, *P* values < 2.2e-16, fig. 2*B*). The pattern of faster-Z evolution was mainly driven by the MBG, which evolved significantly faster on the Z chromosome than on the autosomes (*P* value = 2.1e-4, fig. 2*C*). The results were similar when genes were classified as sex-biased using *P. machaon* adults, but in this case, all classes of genes (MBG, FBG, and UBG) contributed to the faster-Z effect, although neither category was individually significant (supplementary fig. S5, Supplementary Material online).

A faster-Z effect was also observed when sequence divergence was assessed: Z-linked genes had a significantly higher Ka/Ks than autosomal genes (median of 0.1161 vs. 0.0802, *P* value = 1.3e-6, fig. 2*D*). MBG also evolved faster at the sequence level than UBG (*P* value = 9.0e-6), but FBG evolved more slowly than UBG (*P* value = 3.1e-9, fig. 2*E*). Furthermore, UBG showed the strongest faster-Z effect (*P* value = 2.0e-6, fig. 2*F*).

These results confirm that Z-linked genes generally evolve faster than autosomal genes in both their sequence and their expression levels, and that the expression of sex-biased genes also changes rapidly, potentially driving rapid shifts in the sex-biased content of the Z in related species.

### Full Dosage Compensation in Somatic Tissues of *B. mori* and *P. interpunctella*

The extent of dosage compensation varies between tissues and life stages in organisms with well-described compensation mechanisms, such as *Drosophila* (Franke etal. 1996; Meiklejohn and Presgraves 2012; Huylmans and Parsch 2015), and often involves strong sex-biased expression of the sex-chromosomes in gonads (Vicoso and Bachtrog 2015; Gu etal. 2017). The Indian mealmoth *P. interpunctella* has been described to have incomplete, gene-by-gene equalization (Harrison etal. 2012), but these patterns were derived from whole-body expression data, which contains both somatic and gonadal tissue. We therefore sampled *B. mori* and *P. interpunctella* adult heads, thorax, and gonads, as well as *P. interpunctella* larval heads to obtain a comparable overview of the extent of dosage compensation in the two species. RNA-seq library size, number of mapping reads, and number of expressed genes are listed in supplementary tables S1 andS2 in the Supplementary Material online.

Since no genome with linkage information is available for *P. interpunctella*, we followed the approach of Harrison etal. (2012) and defined transcripts as Z-linked and autosomal based on the location of their putative ortholog in *B. mori*, resulting in 303 Z-linked and 7,208 autosomal transcripts. Due to the significant amount of divergence between our *B. mori* samples and the genome reference strain, we assembled the *B. mori* transcriptome de novo from all combined RNA-seq reads and mapped the resulting scaffolds to the reference genome. Of the total 547,645 scaffolds in the de novo assembly, 143,945 could be mapped to 13,514 genes, resulting in 602 Z-linked and 12,183 autosomal genes (the rest remained unassigned). RNA-seq reads were mapped to these gene sets to investigate male/female expression in individual tissues with NextGenMap (Sedlazeck etal. 2013), RPKM values were estimated using custom python scripts, and sex-biased genes were called with DESeq2 (Love etal. 2014).

**Fig. 3.**
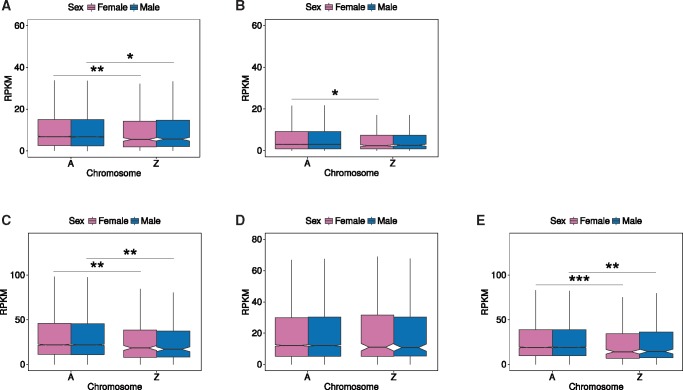
Dosage compensation in somatic tissues of the moths *Bombyx mori* (*A* and *B*) and *Plodia interpunctella* (*C–E*). Only expressed genes were used (RPKM > 0 in both sexes). (*A*) Adult head *B. mori* gene expression on the Z chromosome and the autosomes in males and females. (*B*) Adult thorax *B. mori* gene expression on the Z chromosome and the autosomes in males and females. (*C*) Adult head *P. interpunctella* gene expression on the Z chromosome and the autosomes in males and females. (*D*) Adult thorax *P. interpunctella* gene expression on the Z chromosome and the autosomes in males and females. (*E*) Larva head *P. interpunctella* gene expression on the Z chromosome and the autosomes in males and females. ***P <* *0.001, **P <* *0.01, *P <* *0.05, comparisons without significance stars are nonsignificant (*P *>* *0.05), Wilcoxon rank test.

Z-linked genes were expressed at similar levels in males and females in both *B. mori* and *P. interpunctella* somatic tissues (heads and thorax) indicating that full dosage compensation is present in both species (*P* values > 0.05 in *B. mori* and *P* values > 0.7 in *P. interpunctella*; fig. 3 and table 1). In both adult and larva head (fig. 3*A*, *C*, and *E*), the Z chromosome was further expressed at significantly lower levels than the autosomes in both males and females (*P* values < 0.04, Z:A < 0.84, table 1), but only when low-expression gene were included (this effect disappeared at other RPKM cut-offs in *B. mori* and at RPKM > 5 in *P. interpunctella*; supplementary fig. S6, Supplementary Material online). This is consistent with dosage compensation being at least partially achieved through the down-regulation of Z-linked genes in males, as has been suggested before (Walters and Hardcastle 2011; Smith etal. 2014; Walters etal. 2015; Gu etal. 2017). Contrary to this, we did not detect any down-regulation of Z expression in the male thorax of either species (fig. 3*B* and *D*), irrespective of the expression cut-off (supplementary figs. S6 and S7, Supplementary Material online).

### An Excess of Strongly Testis-Biased Genes on the Z Chromosome Masculinizes Its Expression

**Fig. 4.**
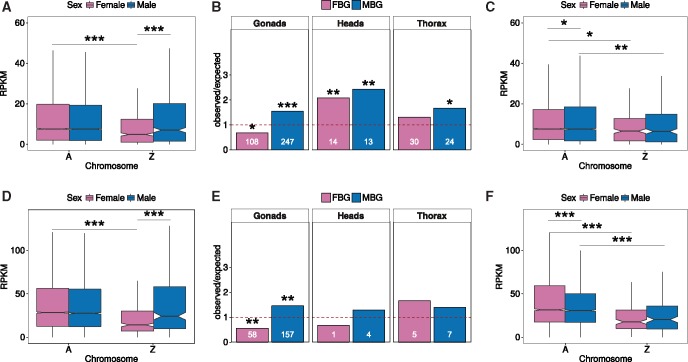
Dosage compensation in gonads of the moths *Bombyx mori* (*A–C*) and *Plodia interpunctella* (*D–F*). Only expressed genes were used (RPKM > 0 in both sexes). (*A*) Adult gonad *B. mori* gene expression on the Z chromosome and the autosomes in males and females. (*B*) Distribution of sex-biased genes in *B. mori* adult tissues on the Z chromosome. (*C*) Adult gonad *B. mori* gene expression on the Z chromosome and the autosomes in males and females excluding sex-biased genes with fold-change > 2. (*D*) Adult gonad *P. interpunctella* gene expression on the Z chromosome and the autosomes in males and females. (*E*) Distribution of sex-biased genes in *P. interpunctella* adult tissues on the Z chromosome. (*F*) Adult gonad *P. interpunctella* gene expression on the Z chromosome and the autosomes in males and females excluding sex-biased genes with fold-change > 2. ***P <* *0.001, **P <* *0.01, *P <* *0.05, comparisons without significance stars are nonsignificant (*P *>* *0.05), Wilcoxon rank test for RPKM values, FET for over-/underrepresentation of sex-biased genes.

In contrast to the results in somatic tissues, which showed full dosage compensation of Z chromosome expression, the expression of Z-linked genes in gonads was reduced in females relative to males in both *B. mori* and *P. interpunctella* (fig. 4*A* and *D*, respectively; *P* value = 4.8e-4, M:F = 1.441 in *B. mori* and *P* value = 4.6e-6, Z:A = 1.709 in *P. interpunctella*). Further, while expression levels of Z-linked genes in males did not differ from those of autosomal genes (*P* values > 0.4, Z:A ratios ∼1), there was reduced gene expression on the Z chromosome relative to the autosomes in females (*P* value = 8.4e-10, Z:A= 0.641 in *B. mori* and *P* value = 2.4e-15, Z:A= 0.499 in *P. interpunctella*, table 1).

Gonads in both *B. mori* and *P. interpunctella* showed a similar enrichment of MBG and depletion of FBG on the Z chromosome as the whole-body data from *P. xuthus*, which is probably dominated by signals from the gonads (fig. 4*B* and *E*). This is different from other tissues, which had much lower numbers of sex-biased genes (fig. 4*B* and *E*;*P. interpunctella* larval heads are not shown because there are no sex-biased genes on the Z chromosome). After excluding sex-biased genes with a fold-change > 2 between males and females from all chromosomes (85 FBG and 211 MBG in *B. mori* and 39 FBG and 128 MBG in *P. interpunctella*, supplementary fig. S8, Supplementary Material online), the expression of Z-linked genes was no longer significantly different between males and females (*P* value = 0.96, M:F = 0.977 in *B. mori* and *P* value = 0.82, M:F = 1.151 in *P. interpunctella*; fig. 4*C* and *F*;table 1). The difference between female expression on the autosomes and the Z chromosome remained significant (*P* value = 0.0038 in *B. mori* and *P* value = 1.6e-10 in *P. interpunctella*), but was accompanied by a similar down-regulation in males (*P* value = 0.035, Z:A = 0.845 in *B. mori* and *P* value = 1.4e-6, Z:A = 0.664 in *P. interpunctella*; fig. 4*C* and *F*;table 1). These patterns were robust to different RPKM cut-offs (supplementary figs. S9 and S10, Supplementary Material online), supporting the presence of chromosome-wide dosage compensation in the gonads once genes with strong sex-biases have been excluded.

## Discussion

### Male-Biased Expression of the Z in Gonads: Absence of Dosage Compensation or a Hotspot for Sex-Biased Expression?

The gonads of both *P. interpunctella* and *B. mori* showed much lower expression of the Z chromosome in females than males. A similar pattern has been observed in *Drosophila melanogaster*, where the X chromosome shows reduced expression in the testis (Vibranovski etal. 2009; Deng etal. 2011). Whether this is due to feminization of the X, absence of dosage compensation in the gonads, or some form of regulatory repression of the X is a hotly debated topic (Gupta etal. 2006; Kemkemer etal. 2011; Meiklejohn etal. 2011; Meiklejohn and Presgraves 2012; Landeen etal. 2016). Dosage compensation also seems to be absent in the germline of other species with XY sex determination (Gartler etal. 1972; Kelly etal. 2002) but the presence of X-inactivation in the male germline makes this difficult to assess in mammals.

Similarly, the masculinized expression of the Z in gonads could be driven by a lack of dosage compensation, by some form of Z-inactivation in the ovary, or by the preferential accumulation of genes with testis-specific functions. The first two models should affect the expression of all genes, while the latter should be reflected mainly in an excess of strongly male-biased genes. Our data is consistent with the last prediction, suggesting that the male-biased expression of the Z can largely be attributed to the functional masculinization of the Z chromosome. However, as the dissected gonads likely contain both meiotic as well as somatic cells, we cannot exclude the possibility that meiotic sex chromosome inactivation (or down-regulation) is present and at least partially contributes to the observed gene expression patterns in lepidopteran gonads. A previous study in *B. mori* using microarrays found a pattern similar to ours: M:F ratios differed significantly between the sexes in gonads but none of the other assayed tissues (Walters and Hardcastle 2011). When the authors excluded all sex-specific genes, this effect was reduced by 25% (Walters and Hardcastle 2011). In the moth *C. pomonella*, however, the exclusion of tissue-specific genes (including testis-specific ones) did not change the authors’ conclusion that dosage compensation is absent in the germline (Gu etal. 2017).

In either case, the excess of male-biased genes on the Z chromosome that we observed in the gonads of *P. interpunctella* likely explains why the initial whole-body study (Harrison etal. 2012) did not yield evidence of dosage compensation. A similar masculinization of the Z or feminization of the X in the gonads may be at play in other species that appear to lack dosage compensation, emphasizing the importance of studying individual tissues (especially in invertebrates, where gonads often comprise a large fraction of the body mass).

### Mechanism of Dosage Compensation: Up- versus Down-Regulation

Gene expression equalization of X/Z chromosomes between the sexes can occur in two (nonexclusive) ways: 1) Up-regulation of the X/Z chromosome in the heterogametic sex, as in *Drosophila* (Kelley etal. 1997; Straub etal. 2005). 2) Down-regulation of expression in the homogametic sex, as observed in mammalian X-inactivation (Lyon 1961). Previous studies in Lepidoptera yielded mixed evidence as to how dosage compensation is achieved in this group: both the silkworm *B. mori* and *Heliconius* butterflies showed lower gene expression on the Z of both sexes than on the autosomes, consistent with down-regulation of this chromosome in males (Walters and Hardcastle 2011; Walters etal. 2015). Male down-regulation of the Z was confirmed at the molecular level in male *B. mori* embryos, where RNAi knockdown of the sex-determination gene *Masc* lead to a global increase in expression of Z-linked genes (Kiuchi etal. 2014). However, the evidence in other species has been less conclusive. Adult heads of the moth *M. sexta* showed slightly but significantly lower Z expression compared with autosomes, but this difference disappeared when low expression genes were excluded from the analysis (Smith etal. 2014). Gu etal. (2017) found that the Z chromosome was down-regulated relative to the autosomes in head, midgut, and ovary tissues of the moth *C. pomonella*, but not in accessory gland or testis.

Similarly, the expression of Z-linked genes was reduced in our *P. interpunctella* and *B. mori* head and gonad data, but only when low expression genes were included. The thorax data showed Z:Autosome ratios of 1 in both species, in apparent agreement with up-regulation of Z expression in the female. However, a high Z:Autosome ratio of expression does not exclude down-regulation in the homogametic sex as a mechanism of equalization, and in fact follows the classic predictions that Ohno (1967) made regarding its origin in mammals: if dosage compensation arises because of imbalances of gene networks that involve sex-linked and autosomal genes in the heterogametic sex, then selection will initially favor an up-regulation of expression, even if it occurs in both sexes (as reduced expression of a whole chromosome is generally more deleterious than excessive expression; Chen ZX etal. 2014). This would leave the homogametic sex with an excess of sex-linked gene expression, secondarily selecting for down-regulation in this sex, and thereby re-establish ancestral levels of expression (and an X/Z:Autosome = 1 in both sexes). Such an evolutionary scenario may also provide an explanation as to why the Z:Autosome ratio depends on expression cut-offs, if the initial up-regulation affected primarily high-expression, dosage sensitive genes, similar to what has been suggested for mammals (Lin etal. 2012; Pessia etal. 2012).

### Differences in the Distribution of Sex-Biased Genes

In three of our four species (*P. xuthus*, *B. mori*, *P. interpunctella*), we found an enrichment of genes with male/testis-biased expression on the Z chromosome. This is in agreement which theoretical studies that predict that genes encoding for traits that are under sexual selection, as may be expected of those with functions in the germline (testis-specific and testis-biased genes; Ellegren and Parsch 2007), should accumulate on the Z chromosome (Reeve and Pfennig 2003; Albert and Otto 2005). More generally, since they spend 2/3 of their time in the homogametic sex, both Z and X chromosomes are expected to accumulate sexually antagonistic mutations that benefit this sex, as long as these mutations are dominant (for recessive mutations, the opposite is expected; Rice 1984). This could potentially lead to the acquisition of male-specific functions for many genes on the Z chromosome, and their consequent male-biased or male-specific patterns of expression (Rice 1984). Differential distribution of sex-biased genes on sex chromosomes has been found in various clades (Reineke etal. 2000; Parisi etal. 2003; Kaiser and Ellegren 2006; Storchová and Divina 2006; Arunkumar etal. 2009; Reinius etal. 2012; Jaquiéry etal. 2013; Vicoso, Kaiser, etal. 2013; Pal and Vicoso 2015; Vicoso and Bachtrog 2015; Gu etal. 2017), and generally shows an enrichment for genes expressed primarily in the homogametic sex (but see Wang etal. 2001 and Lercher etal. 2003 for counterexamples in mammals).

Consistent with this, several Lepidoptera studies of either abdomen or gonad detected an enrichment of male-biased or male-specific genes and depletion of female-biased genes on the Z chromosome (Arunkumar etal. 2009; Walters etal. 2015; Gu etal. 2017). Contrary to this, despite using whole-body data, which should contain gonads, we found no masculinization of the Z chromosome in adult *P. machaon* (and in fact detected an excess of female-biased genes); this is in contrast with the results for the closely related to *P. xuthus*. Two caveats should be mentioned regarding these results: first, only one replicate was available for each sex, so that this difference could simply reflect biological noise. Second, the proportion of the adult body taken up by gonads may change during the lifetime, so that different individuals may simply not carry sufficient gonadal tissue for the masculinization of the Z to be apparent from whole-body data. However, it is also possible that these results reflect an actual biological difference between the two species. In particular, while *P. xuthus* females commonly mate with multiple males during their lifetime, *P. machaon* females tend to be monandrous (Wantanabe and Nozato 1986; Wantanabe and Kobayashi 2006). Sexual selection, especially in the form of sperm competition, should therefore be greatly reduced in the second species. In birds, reduced evidence of sexual selection is associated with overall decreased amounts of male-biased expression in the genome (Harrison etal. 2015). Reduced strength of sexual selection may similarly have lead to the demasculinization of the Z chromosome in the monandrous *P. machaon*; further studies of mon- and polyandrous Lepidoptera species will in the future allow for a direct test of the association between strong sexual selection and functional masculinization of the Z chromosome.

### The Difference between Lepidoptera and Other ZW Clades

While it is now clear that lepidopteran insects are exceptional among ZW clades in having complete and widespread dosage compensation, why such chromosome-wide mechanisms evolved in this group but not in the others is not well understood. In XY systems, species with young sex chromosomes also often lack chromosome-wide dosage compensation (Schultheiß etal. 2015), and one possible explanation would be a difference in age between the lepidopteran Z chromosome and that of other groups; younger Z chromosomes may simply not have had enough time to acquire chromosome-wide compensation. Female-heterogamety likely evolved in the common ancestor of Lepidoptera and its sister order Trichoptera and thus the Z chromosome may be close to 200 My old (Sahara etal. 2012). The sex chromosomes of birds and snakes, the other clades with ZW sex determination that have been studied in more detail and for which well resolved and timed phylogenies are available, are younger at ∼150 and 100 My old, respectively (Nam and Ellegren 2008; Matsubara etal. 2016). However, XY chromosomes of similar ages are typically compensated.

Another factor in which Lepidoptera differ from other investigated ZW systems is their effective population size (N_e_). Insects generally have a larger N_e_ than vertebrates (such as snakes and birds), and this can strongly influence patterns of adaptive evolution. Mullon etal. (2015) showed that the small N_e_ of the Z chromosome reduces the probability of fixation of compensatory mutations that increase the expression of haploid Z-linked genes. Under this model, only Z-linked genes under strong selection for optimal dosage become dosage compensated (Mullon etal. 2015). However, since the proportion of mutations that are effectively neutral results from a combination of the general N_e_ of the species and the Z-specific reduction in N_e_, many more mutations that increase expression should be under effective positive selection in Lepidoptera than in ZW vertebrates, potentially leading to more widespread dosage compensation.

Finally, the lack of meiotic recombination observed in females of many Lepidopteran species (Marec 1996) may have been ancestrally present and lead to fast degeneration of the original W chromosome, creating a strong selection pressure on females to compensate for the hemiploidy of Z-linked genes. The more gradual decay of the W chromosome in birds and snakes (where females recombine, and some lineages have largely undifferentiated ZW pairs), may have allowed for the evolution of gene-by-gene regulation for the few dosage-sensitive genes lost on the W, rather than requiring a full chromosome-wide mechanism. These different hypotheses are not mutually exclusive, and understanding their relative contributions will require the assessment of dosage compensation in ZW and XY species with a variety of life history and population genetics parameters.

### Conclusion

We found equal expression of the Z chromosome in males and females in multiple developmental stages and tissues of four different Lepidoptera species consistent with widespread Z chromosome-wide dosage compensation in this clade. Furthermore, our data indicate that the accumulation of sex-biased genes, especially in the germline, can mask these patterns and should be taken into account when assessing dosage compensation in other species. Our findings of full dosage compensation in Lepidoptera raise the question of whether the presence of Z chromosome-wide dosage compensation in other ZW species may have been overlooked previously due to masculinization of the Z chromosome and shed new light on the interplay between the selective pressures to maintain balanced expression on the sex chromosomes and sex-specific selection.

## Materials and Methods

### Sampling

*Bombyx mori* eggs were ordered from silkwormstore.co.uk. Larvae were hatched in the lab at room temperature (∼24 °C) and fed at libitum with powdered mulberry leaves, reconstituted with water. Wild *P. interpunctella* were caught in Vienna, Austria in the summer of 2015 and kept on a diet of mixed nuts at 21–22 °C on a 10:14 hours light:dark cycle.

*Plodia interpunctella* 5th instar larvae were sexed based on the presence/absence of the testes visible through the cuticula. Upon eclosure, adults were sexed based on genitalia structures. *Plodia interpunctella* 5th instar larvae were sacrificed directly, dissected in 1× PBS buffer and the tissue was then disrupted in RTL lysis buffer (QIAGEN, Hilden, Germany). Two- to five-day-old *P. interpunctella* unmated adults were anesthetized using CO_2_. Adult heads and thorax were severed from the rest of the carcass and directly placed into chilled RTL lysis buffer. Adult gonads were first dissected in 1× PBS buffer and then disrupted in RTL lysis buffer. For each *P. interpunctella* sample, tissue from five individuals was pooled.

*Bombyx mori* adults were also sexed directly after eclosion based on genitalia. Unmated adults were sacrificed 24 h after eclosion and were anesthetized by placing them at 4 °C for 15 min prior to dissection. Adult gonads were also dissected in 1× PBS buffer and all tissues (adult heads, gonads, and thorax) were placed into cold RTL buffer for tissue disruption. In contrast to *P. interpunctella*, tissue from only one *B. mori* individual was used per sample. For all samples the tissue was disrupted using a pestle, frozen in liquid nitrogen and stored at −80 °C until RNA extraction.

### RNA Preparation and Sequencing

RNA extractions were performed using the Qiagen RNeasy kit (QIAGEN, Hilden, Germany) according to the manufacturer’s instructions. The RNA concentration was assessed using a NanoDrop spectrophotometer (Thermo Scientific, Wilmington, DE) and the quality was subsequently checked on a Bioanalyzer 2100 (Agilent Technologies, Santa Clara, CA). For both species, two high-quality biological replicates per sex and tissue were chosen for sequencing. Library preparation and sequencing were performed by the VBCF NGS Unit (www.vbcf.ac.at, Vienna, Austria). Samples were sequenced on an Illumina HiSeq2500 on a total of three lanes producing 125 bp paired-end (PE) reads with an average insert size of 160 bp. Sequencing statistics can be found in supplementary tables S1 and S2 in the Supplementary Material online for *B. mori* and *P. interpunctella*, respectively. The quality of the sequenced libraries was verified with fastQC (version 0.11.5, Andrews 2010).

### Gene Expression

For *B. mori* and *P. interpunctella*, we used our own RNA-seq data sets (see above), while for *P. xuthus* and *P. machaon*, published RNA-seq data for a single male and female adult, as well as male and female pupa were downloaded from Gene Expression Omnibus (accessions SRR1760413–SRR1760416 for *P. xuthus* and SRR1760417–SRR1760420 for *P. machaon*, Li etal. 2015).

Coding sequences for *P. xuthus* and *P. machaon* (Li etal. 2015, version 1.0 in both cases), as well as the chromosomal location in *P. xuthus*, were obtained from ftp://ftp.genomics.org.cn/pub/papilio. The transcriptome assembly of *P. interpunctella* (Harrison etal. 2012) was provided by Peter Harrison. We used the published transcriptome rather than the genome, which has not been published yet but is available on Lepidoptera Base (http://ensembl.lepbase.org/Plodia_interpunctella_v1/Info/Index), because there is no linkage information for the genome and to make our analyses comparable to those of Harrison etal. (2012). *Plodia interpunctella* transcripts (Harrison etal. 2012) were first mapped to the whole set of *B. mori* protein sequences (SilkDB, genome version 2.0, Wang etal. 2005; International Silkworm Genome Consortium 2008) using Blast (Altschul etal. 1990) with an e-value cut-off of e-5. The reverse mapping was then performed (*B. mori* proteins against *P. interpunctella* transcripts) with the same parameters and bi-directional best hits (BBH) were extracted to identify 1-to-1 orthologs. Only *P. interpunctella* transcripts with a 1-to-1 ortholog in *B. mori* were retained for further analysis.

As the *B. mori* strain for which the genome was published (version 2.0, International Silkworm Genome Consortium 2008) differed significantly from ours (only ∼48% of RNA-seq reads mapped to the genome using NextGenMap, Sedlazeck etal. 2013), we reassembled the *B. mori* reads from our combined samples using SOAPdenovo-Trans (version 1.03, Xie etal. 2014) paired-end mode with multiple k-mers (21–81 bp, step size of 10). The assembly with a k-mer length of 51 bp was chosen for further analysis and all scaffolds smaller than 300 bp were discarded. As this assembly was highly fragmented compared with the gene models from the genome version (an average of 10 scaffolds map to each gene model) we allowed multiple scaffolds to map to the same *B. mori* reference gene model (but not to multiple gene models) and excluded all unmapped scaffolds from further analyses. Scaffolds that mapped to the same *B. mori* gene model were considered to be part of a single gene in our downstream analysis (see below).

Genes assigned to the Z chromosome in the respective genome papers of *P. xuthus* and *B. mori* were used as the Z-linked data set, while genes assigned to other chromosomes were designated as autosomal genes. Those on unassigned scaffolds were excluded from the downstream analysis. Since no linkage information is available for *P. machaon* or *P. interpunctella*, we relied on the chromosomal location of the *P. xuthus* and *B. mori* 1-to-1 orthologs obtained with the bi-directional best hit blast, respectively, as the Z chromosome is highly conserved among Lepidoptera (Pringle etal. 2007; Ahola etal. 2014). However, as there have been some rearrangements on the *Papilio* Z chromosome in comparison to the *B. mori* Z chromosome (Li etal. 2015), we also blasted *P. xuthus* and *P. machaon* protein sequences against *B. mori* proteins and vice versa to obtain 1-to-1 orthologs between *B. mori* and the two *Papilio* species. These were used to infer which genes are part of the conserved Z chromosome (termed old) and which are located on other chromosomes in *B. mori* (termed new) and test whether this evolutionary age could influence the assessment of the dosage compensation status.

RNA-seq reads were aligned to the coding-sequence (CDS) of the respective species using NextGenMap (version 0.4.12, Sedlazeck etal. 2013). Gene expression values (in Reads Per Kilobase of transcript per Million mapped reads, RPKM) were calculated for each gene in each of the RNA-seq libraries with custom python scripts. Published RPKM values were already available for *P. xuthus* and *P. machaon* (obtained with TopHat, Li etal. 2015). Since these yield very similar results to our own RPKM estimates, we only show the latter, which are more appropriate for direct comparisons between the four species. Comparisons of expression of the Z chromosome versus the autosomes in males and females were performed after applying a quantile normalization with the Bioconductor package “preprocessCore” (version 1.32.0, Gentleman etal. 2004; Bolstad 2017) as implemented in R (version 3.1.1, R Core Team 2015) to normalize between libraries in each tissue and developmental stage. Dosage compensation was assessed at four different levels: without any expression cut-off, with RPKM > 0, RPKM > 1, and RPKM > 5 across all libraries of the respective tissue and developmental stage. These RPKM values were then used to compare expression of the Z chromosome and the autosomes in males and females and for the calculation of Z-to-autosome ratios (Z:A) and male-to-female ratios (M:F).

### Sex-Biased Genes

The Bioconductor package DESeq2 (version 1.10.1, Love etal. 2014), as implemented in R, was used to identify genes that are differentially expressed between the sexes for the different tissues and developmental stages of *B. mori* and *P. interpunctella*. Raw read counts resulting from the NextGenMap mappings were used as the input for DESeq2. A Benjamini–Hochberg correction (implemented in DESeq2) was used to adjust for multiple testing (Benjamini and Hochberg 1995). All genes with a significant adjusted *P* value < 0.05 were considered to be sex-biased. As there were no replicates for *P. xuthus* and *P. machaon*, it was not feasible to perform the same differential expression analysis in these species. Instead, differentially expressed genes between the sexes were determined using GFOLD (version 1.1.4, Feng etal. 2012). Only genes with a fold-change > 2 were considered sex-biased. All genes that did not meet this criterion were considered to be unbiased. The degree of sex bias of each gene is measured as the log2 fold-change between male and female expression.

To test whether sex-biased genes on the Z chromosome deviated from random distribution, we calculated the ratio of observed to expected number of male- and female-biased genes. This was done for each tissue and developmental stage separately. The expected number of MBG or FBG was obtained via multiplication of the proportion of Z-linked genes in the whole data set (depending on the RPKM cut-off, see above) with the total number of MBG or FBG. A two-sided Fisher’s exact test was used to check whether MBG andFBG deviated from random distribution. Whenever sex-biased genes were accounted for in the analysis of the dosage compensation status, we excluded MBG and FBG with a fold-change > 2 (or 4 where indicated) from all autosomes, as well as the Z chromosome.

### Faster-Z Evolution

To compare rates of evolution on the Z and autosomes, we calculated both the rate of gene expression change, as well as rates of sequence evolution between the sister species *P. xuthus* and *P. machaon*. The moths *P. interpunctella* and *B. mori* are too distantly related for a similar analysis to be meaningful, and were therefore not included.

To quantify the change in gene expression between *P. xuthus* and *P. machaon*, we calculated the Euclidean distance between them (following the approach of Pereira etal. 2009) for all 10,803 1-to-1 orthologs with a known chromosomal location in *P. xuthus*. Males and females, as well as the two different stages (adult and pupa) were treated as different tissues for this calculation. For the classification of sex-biased genes, *P. xuthus* and *P. machaon* adults were used separately, to check that our results held when the two different sets of genes were considered. Figures are based on the classification obtained for *P. xuthus* adults, but figures using the *P. machaon* adult sex-biased biased genes instead are provided in supplementary fig. S5 in the Supplementary Material online. Euclidean distances for groups of genes were compared using Wilcoxon rank tests.

The rate of synonymous (Ks) and nonsynonymous (Ka) sequence divergence between *P. xuthus* and *P. machaon* was calculated for each of the 1-to-1 orthologs. For this, protein sequences of orthologous genes were first aligned with MUSCLE (Edgar 2004) and these protein sequence alignments were used to produce codon-aware nucleotide alignments with tranalign (Rice etal. 2000). Ka and Ks values were then calculated for each gene alignment with KaKs_Calculator (version 2.0, Wang etal. 2010) using default parameters. Ka/Ks values were compared between groups of genes using Wilcoxon rank tests.

## Supplementary Material

Supplementary data are available at *Molecular Biology and Evolution* online.

## Supplementary Material

Supplementary Data
