# Supplementary material for: Global Dosage Compensation Is Ubiquitous in Lepidoptera, but Counteracted by the Masculinization of the Z Chromosome
Source: Mol Biol Evol. 2017 Jul 6;34(10):2637–49. doi: 10.1093/molbev/msx190 (PMC5850747; doi:10.1093/molbev/msx190)
Supplement: Supplementary Data [file msx190_supp.pdf]

Supplementary Material: Global dosage compensation is ubiquitous in Lepidoptera, but counteracted by the masculization of the Z chromosome

Ann Kathrin Huylmans, Ariana Macon, and Beatriz Vicoso

Table S1: *B. mori* sequencing libraries, mapping reads and numbers of expressed genes per tissue used for analyses (some genes are located on unassigned scaffolds).

Sample	Tissue	Sex	Stage	Library Size	Mapping Reads	Expressed Genes (RPKM>0)
37854	Head	M	Adult	49,253,204	38,629,298	12,410 (Z: 560; A: 11,193)
37855	Head	M	Adult	50,536,958	39,709,994	
39188	Head	F	Adult	51,994,070	40,853,461	
39189	Head	F	Adult	56,590,302	44,175,726	
39186	Thorax	M	Adult	60,562,878	51,853,507	11,748 (Z: 510; A: 10,649)
39187	Thorax	M	Adult	59,314,454	50,284,945	
39190	Thorax	F	Adult	56,562,812	47,900,990	
39191	Thorax	F	Adult	56,390,370	47,738,948	
34791	Gonads	M	Adult	48,528,332	37,882,997	11,783 (Z: 515; A: 10,621)
34792	Gonads	M	Adult	51,610,086	40,209,804	
39192	Gonads	F	Adult	53,451,198	42,694,738	
39193	Gonads	F	Adult	55,746,938	44,320,670	

Table S2: *P. interpunctella* sequencing libraries, mapping reads and numbers of expressed genes per tissue used for analyses (some genes are located on unassigned scaffolds).

Sample	Tissue	Sex	Stage	Library Size	Mapping Reads	Expressed Genes (RPKM>0)
34795	Head	M	Adult	51,020,628	30,206,005	7,420 (Z: 298; A: 6,787)
34796	Head	M	Adult	45,016,890	27,426,265	
34801	Head	F	Adult	50,968,990	30,517,658	
34802	Head	F	Adult	53,106,612	31,062,412	
34797	Thorax	M	Adult	42,454,698	26,332,463	7,372 (Z: 291; A: 6,746)
34798	Thorax	M	Adult	48,620,638	29,640,893	
34803	Thorax	F	Adult	51,852,062	32,089,716	
34804	Thorax	F	Adult	53,119,172	32,561,785	
34799	Gonads	M	Adult	54,213,342	28,829,835	7,424 (Z: 297; A: 6,787)
34800	Gonads	M	Adult	47,904,454	26,988,226	
34805	Gonads	F	Adult	49,898,928	32,884,771	
34806	Gonads	F	Adult	52,892,328	34,399,402	
34789	Head	M	Larva	47,338,792	28,032,388	7,421 (Z: 297; A: 6,788)
34790	Head	M	Larva	49,287,446	30,799,218	
34793	Head	F	Larva	48,356,670	30,700,289	
34794	Head	F	Larva	38,430,562	19,253,655	

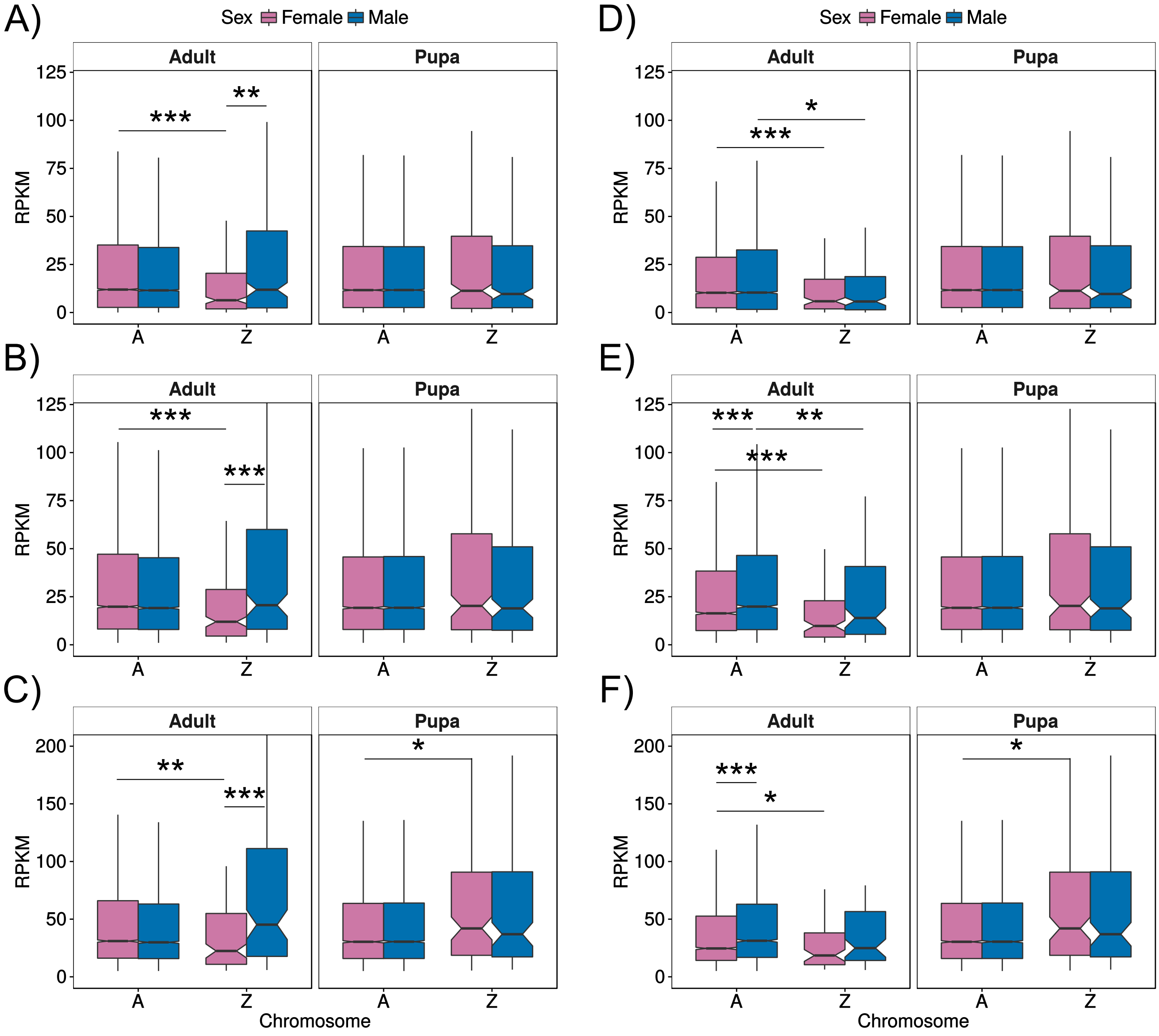

Figure S1: Dosage compensation in *P. xuthus* at different RPKM cut-offs including (A-C) and ecluding (D-F) sex-biased genes. A, D) All genes independent of expression level. B, E) Genes with RPKM>1 in both sexes. C, F) Genes with RPKM>5 in both sexes. $***P < 0.001$, $**P < 0.01$, $*P < 0.05$, comparisons without significance stars are non-significant ($P > 0.05$), Wilcoxon rank test.

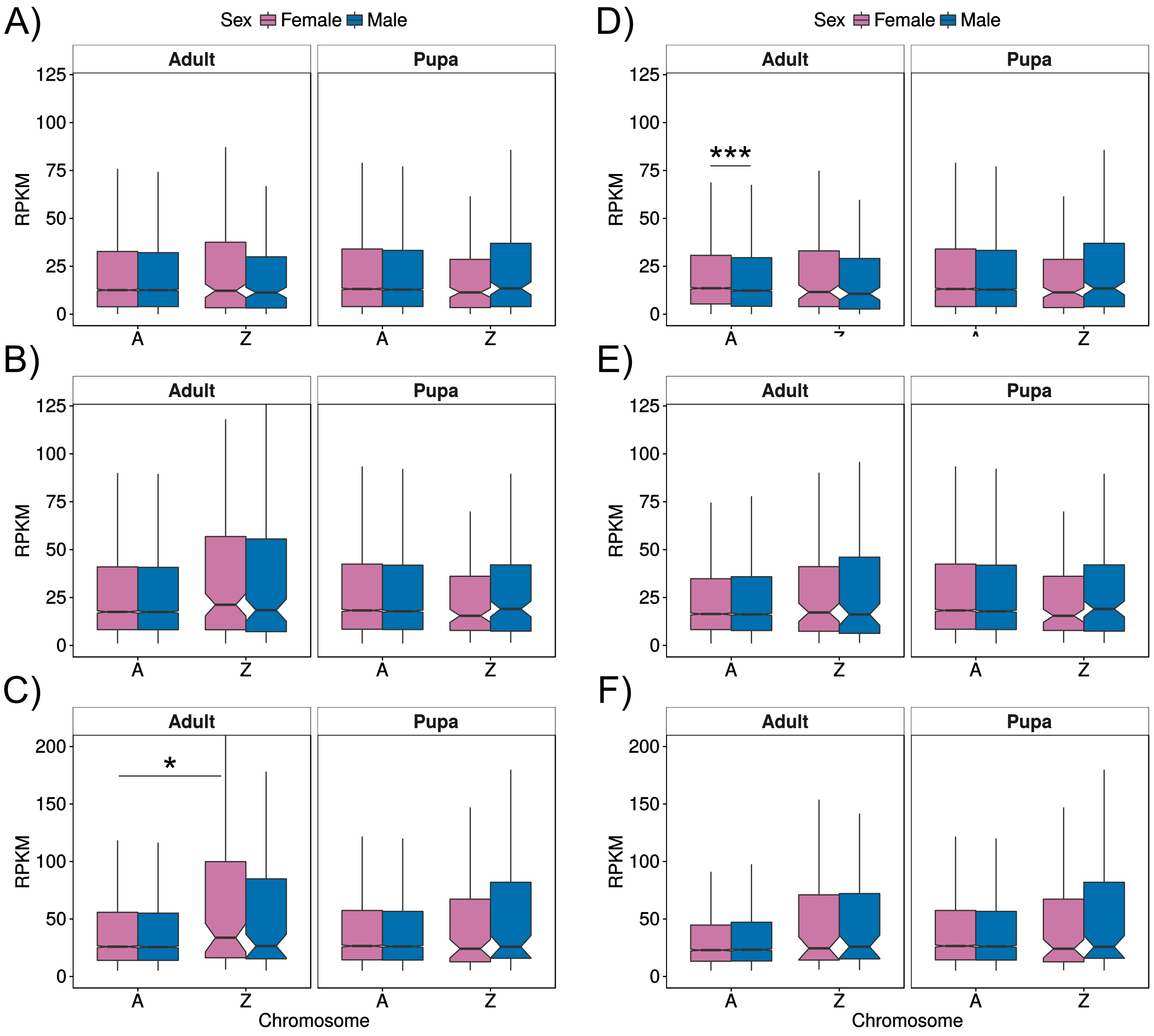

Figure S2: Dosage compensation in *P. machaon* at different RPKM cut-offs including (A-C) and ecluding (D-F) sex-biased genes. A, D) All genes independent of expression level. B, E) Genes with RPKM>1 in both sexes. C, F) Genes with RPKM>5 in both sexes. $***P < 0.001$, $**P < 0.01$, $*P < 0.05$, comparisons without significance stars are non-significant ($P > 0.05$), Wilcoxon rank test.

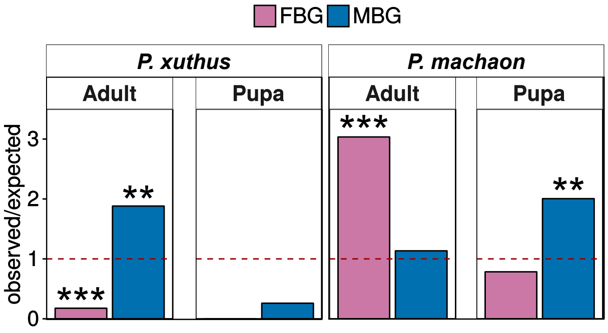

Figure S3: Distribution of highly sex-biased genes (fold-change>4) on the Z chromosomes of two *Papilio* species calculated as the number of observed genes over the number of expected ones for adults and pupae. $***P < 0.001$, $**P < 0.01$, $*P < 0.05$, comparisons without significance stars are non-significant ($P > 0.05$), FET.

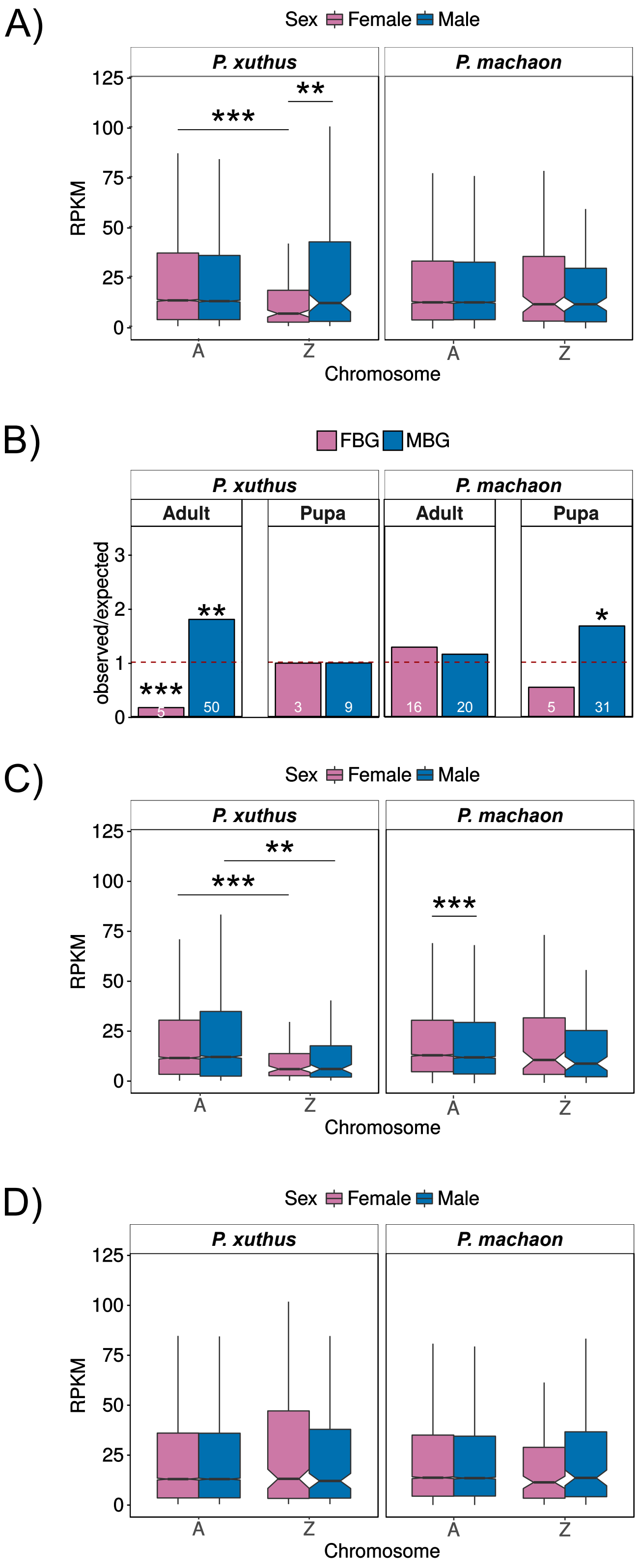

Figure S4: Dosage compensation in *Papilio* butterfly species for expressed genes (RPKM>0 in both sexes) and only considering the conserved part of the Z chromosome (i.e. genes with 1-to-1 orthologs on the *B. mori* Z). A) Adult expression on the old Z chromosome and the autosomes compared in males and females. B) Distribution of sex-biased genes on the old Z chromosome calculated as the number of observed genes over the number of expected ones. Absolute gene numbers are stated in bars. C) Adult expression on the old Z chromosome and the autosomes compared in males and females excluding sex-biased genes. D) Pupa expression on the old Z chromosome and the autosomes compared in males and females. $***P < 0.001$, $**P < 0.01$, $*P < 0.05$, comparisons without significance stars are non-significant ($P > 0.05$), Wilcoxon rank test for RPKM values, FET for over-/under-representation of sex-biased genes.

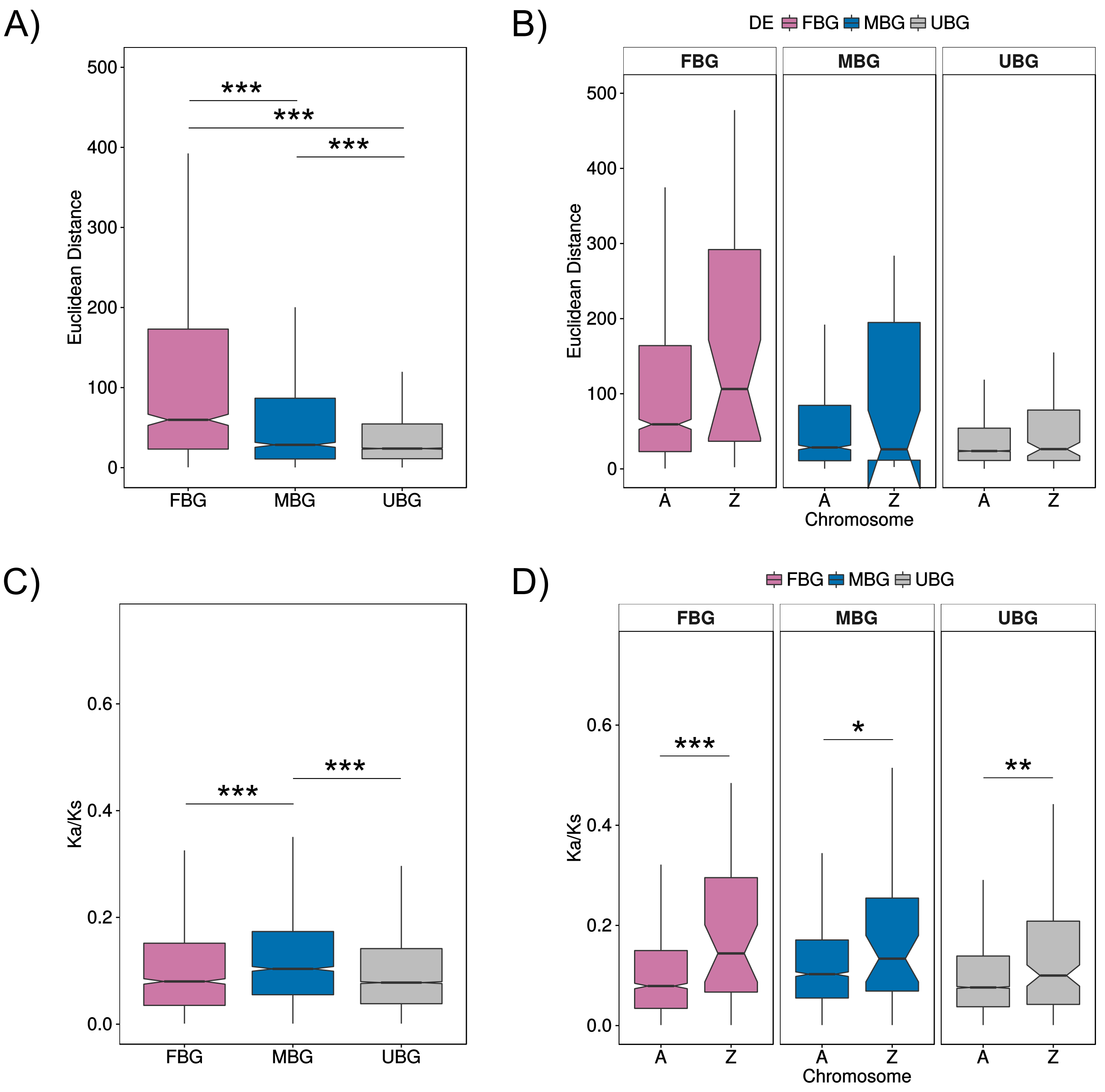

Figure S5: Evolutionary rates of sex-biased genes and Z-linked genes of *Papilio* butterflies. Expression divergence: A) Euclidean distance in expression for male-biased, female-biased, and unbiased genes. B) Euclidean distance in expression for sex-biased genes and chromosomal location (Z chromosome vs autosomes). Sequence divergence: C) Ka/Ks ratios for sex-biased genes. D) Ka/Ks ratios for for sex-biased genes and chromosomal location. Classification of sex-biased genes is based on *P. machaon* adults. $***P < 0.001$, $**P < 0.01$, $*P < 0.05$, comparisons without significance stars are non-significant ($P > 0.05$), Wilcoxon rank test.

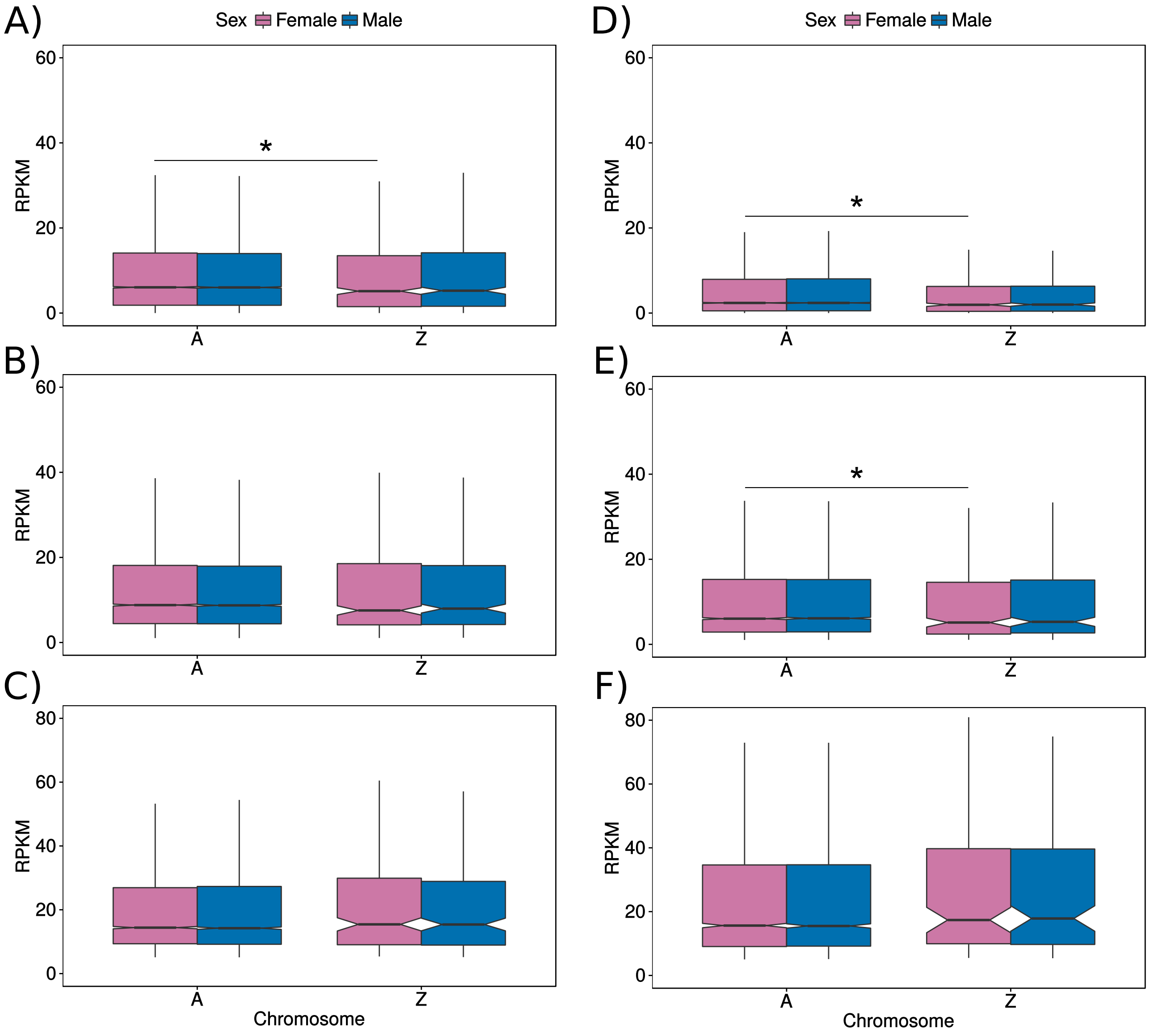

Figure S6: Dosage compensation in somatic *B. mori* tissues at different RPKM cut-offs. A-C) Adult heads, D-F) Adult thorax. A, D) All genes independent of expression level. B, E) Genes with RPKM>1 in both sexes. C, F) Genes with RPKM>5 in both sexes. ***$P < 0.001$, **$P < 0.01$, *$P < 0.05$, comparisons without significance stars are non-significant ($P > 0.05$), Wilcoxon rank test.

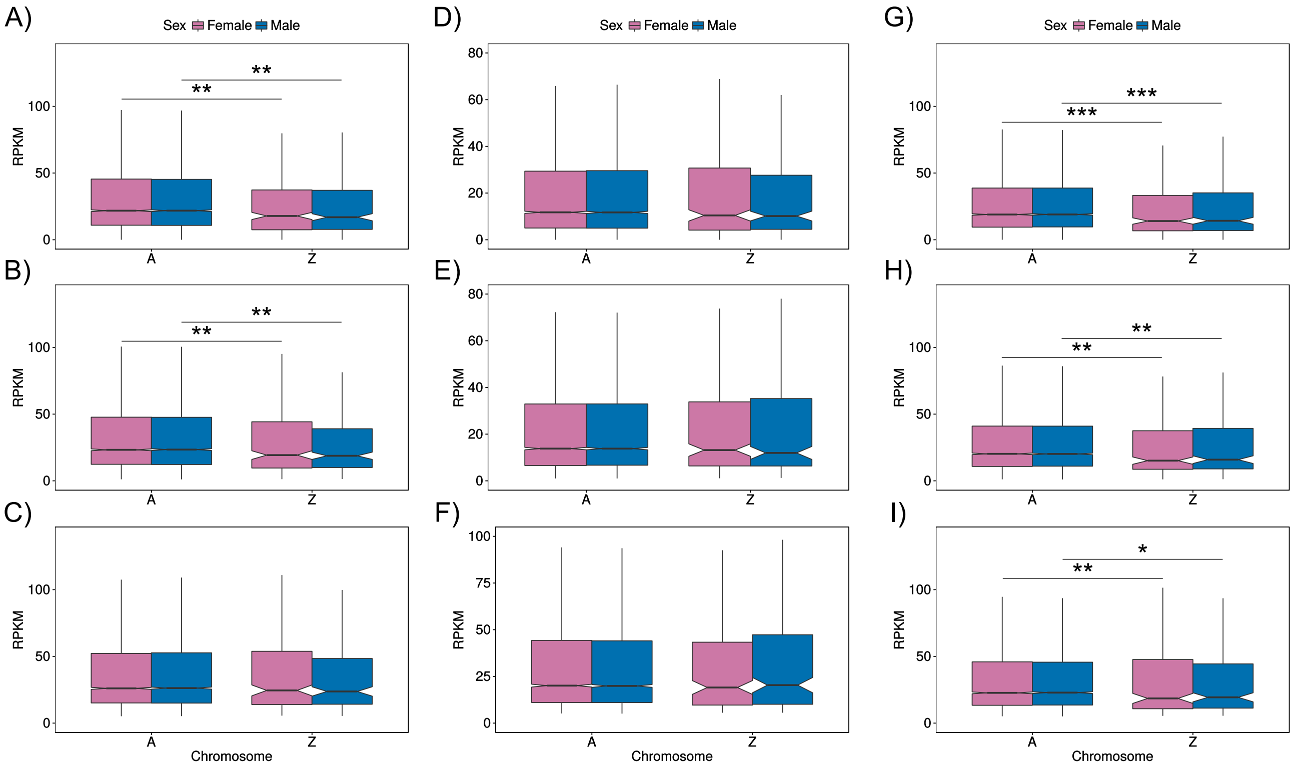

Figure S7: Dosage compensation in somatic *P. interpunctella* tissues at different RPKM cut-offs. A-C) Adult heads, D-F) Adult thorax, G-I) Larva heads. A, D, G) All genes independent of expression level. B, E, H) Genes with RPKM>1 in both sexes. C, F, I) Genes with RPKM>5 in both sexes. $***P < 0.001$, $**P < 0.01$, $*P < 0.05$, comparisons without significance stars are non-significant ($P > 0.05$), Wilcoxon rank test.

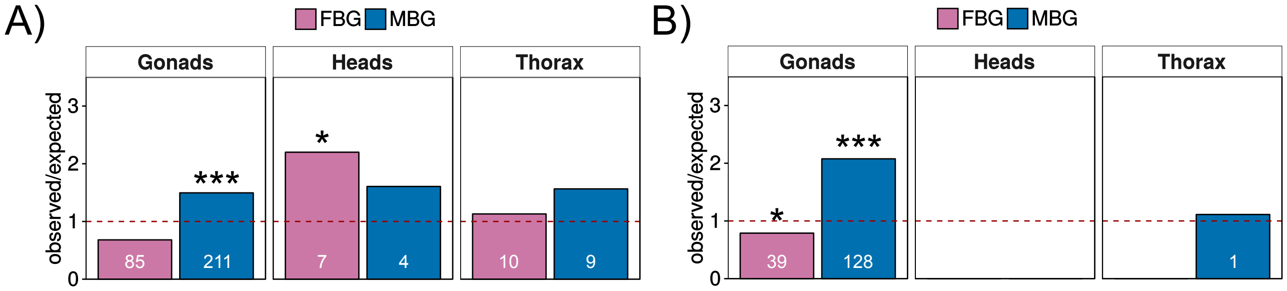

Figure S8: Sex-biased genes on the Z chromosome with fold-change>2 that cannot be explained by lack of dosage compensation. A) Over-/underrepresentation of male-biased (MBG) and female-biased (FBG genes) on the Z chromosome in different adult *B. mori* tissues. B) Over-/underrepresentation of male-biased (MBG) and female-biased (FBG genes) on the Z chromosome in different adult *P. interpunctella* tissues. Numbers below the bar represent number of FBG or MBG on the Z over total number of FBG or MBG expressed in this tissue. $***P < 0.001$, $**P < 0.01$, $*P < 0.05$, comparisons without significance stars are non-significant ($P > 0.05$), FET.

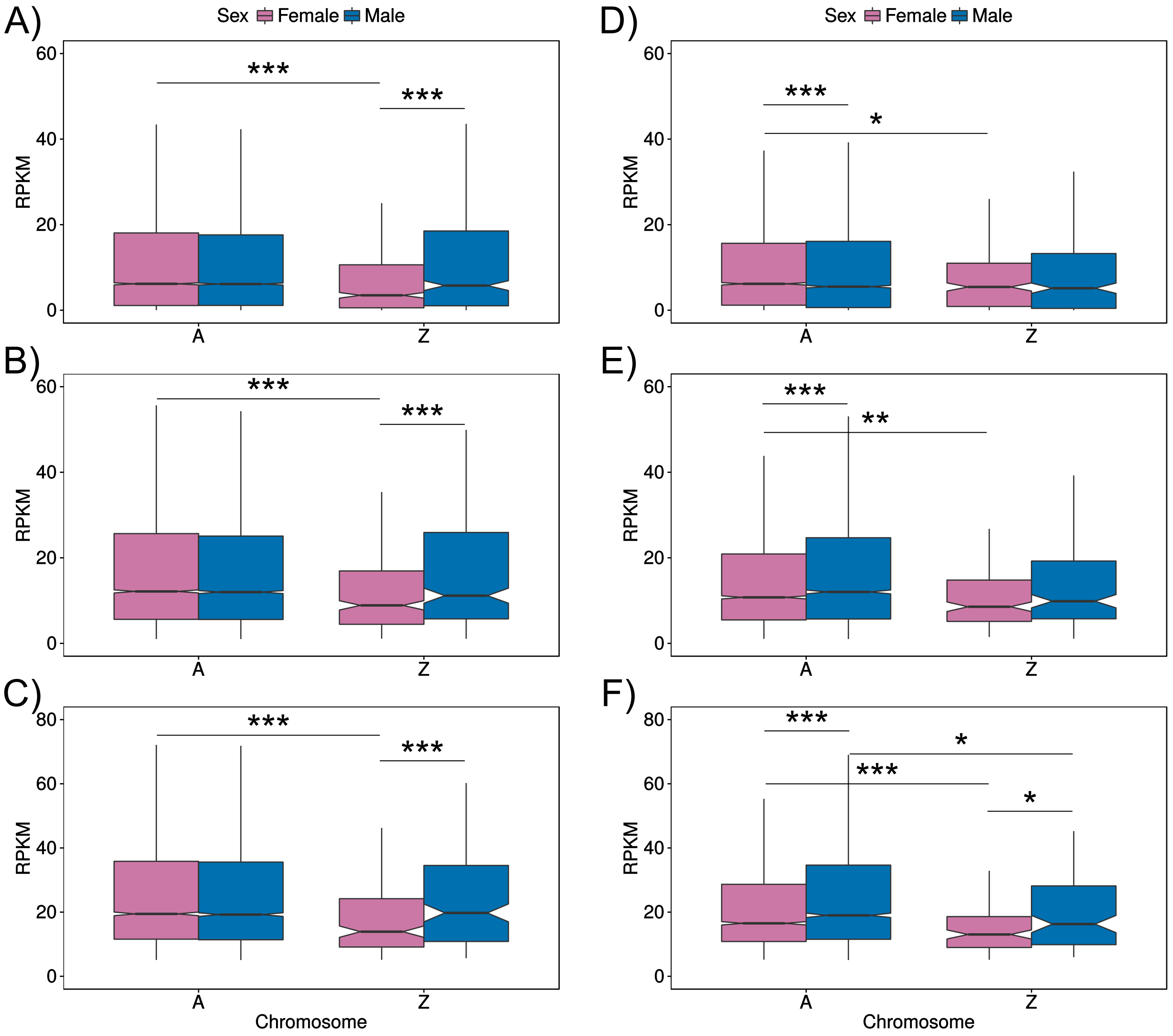

Figure S9: Dosage compensation in *B. mori* gonads at different RPKM cut-offs. A-C) Including sex-biased genes, D-F) excluding sex-biased genes with fold-change>2. A, D) All genes independent of expression level. B, E) Genes with RPKM>1 in both sexes. C, F) Genes with RPKM>5 in both sexes. ***$P < 0.001$, **$P < 0.01$, *$P < 0.05$, comparisons without significance stars are non-significant ($P > 0.05$), Wilcoxon rank test.

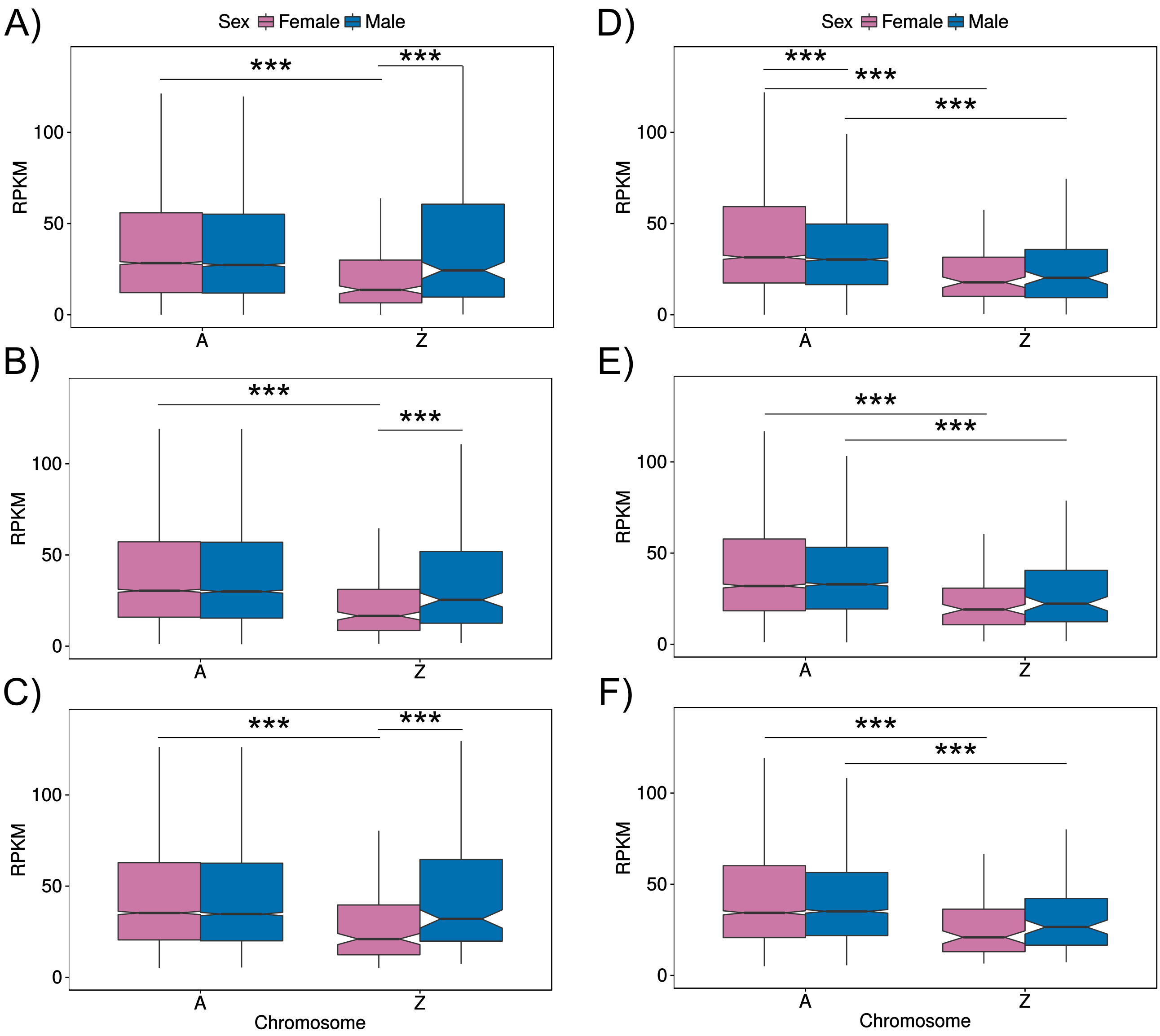

Figure S10: Dosage compensation in *P. interpunctella* gonads at different RPKM cut-offs. A-C) Including sex-biased genes, D-F) excluding sex-biased genes with fold-change>2. A, D) All genes independent of expression level. B, E) Genes with RPKM>1 in both sexes. C, F) Genes with RPKM>5 in both sexes. $***P < 0.001$, $**P < 0.01$, $*P < 0.05$, comparisons without significance stars are non-significant ($P > 0.05$), Wilcoxon rank test.
